# Isolation, Identification, and Pathogenicity of *Aeromonas veronii* from Diseased *Paracobitis variegatus*

**DOI:** 10.3390/ijms27219776

**Published:** 2026-10-30

**Authors:** Zhiqiang Zhang, Yiling Zhou, Qing Zhou, Ying Wang, Changlun Xiao, Dayong Xu

**Affiliations:** 1 Key Laboratory of Application of Ecology and Environmental Protection in Plateau Wetland of Sichuan, Xichang University, Xichang 415000, China; zzq248798423@gmail.com (Z.Z.);; 2 Key Laboratory of Animal Disease Detection and Prevention in Panxi District, Xichang University, Xichang 415000, China; 3 Institute of Animal Husbandry and Fisheries, Yibin Academy of Agricultural Sciences, Yibin 644600, China

**Keywords:** *Aeromonas veronii*, *Paracobitis variegatus*, isolation and identification, pathogenicity

## Abstract

*Aeromonas veronii* (*A. veronii*) is ubiquitous in aquatic habitats and infects diverse aquatic animals, causing high mortality and heavy economic losses in aquaculture. In this study, we initially isolated a Gram-negative bacterium (Pv001) from the visceral organs of naturally diseased *Paracobitis variegatus*. Based on morphological, biochemical, and 16S rRNA and gyrB gene sequence analysis, the isolated strain was accurately determined to be *A. veronii*. Virulence gene screening showed that the strain Pv001 carried multiple virulence genes, including *Acg*, *Act*, *Alt*, *Exu*, *Lip*, and *Omp*. In vitro antimicrobial evaluations revealed that ceftriaxone, chloramphenicol, ciprofloxacin, and tetracycline displayed significant inhibitory effects on Pv001, while ampicillin, penicillin, and lincomycin exhibited no detectable inhibitory action. The artificial challenge of *Paracobitis variegatus* with the isolated strain Pv001 *via* intraperitoneal injection reproduced the clinical signs observed in the affected farm, yielding a calculated LD_50_ of 8.61 × 10^5^ CFU/fish. Histopathological analysis of the diseased fish indicated extensive intestinal mucosal destruction, vacuolar degeneration of liver cells, and localized necrosis, as well as a considerable number of melano-macrophage centers (MMCs) aggregating in the spleen. This study provides a scientific foundation for the prevention and control of epidemics by revealing the pathogenic features of *A. veronii* illness in *P. variegatus*.

## 1. Introduction

*Paracobitis variegatus* (*P. variegatus*) belongs to the order *Cypriniformes*, family *Cobitidae*, and genus *Paracobitis*, and is an endemic and valuable freshwater fish species predominantly distributed in the upper reaches of the Yangtze River in southwestern China [1,2]. It is widely favored in regional markets due to its palatable meat and excellent nutritional properties [2]. Changes in global environmental conditions and human pollutants have altered the living environments of fish populations, resulting in commensurate changes in survival, behavior, and movement [3]. Consequently, in the aquaculture industry, outbreaks of bacterial diseases have become increasingly frequent and catastrophic, severely threatening the sustainable development of this industry [4]. Among these diseases, bacterial septicemia is one of the most common and devastating syndromes, clinically characterized by systemic cutaneous hemorrhaging, abdominal distension, and massive fluid accumulation (ascites) in the coelomic cavity, ultimately leading to high mortality [5,6].

*Aeromonas veronii* (*A. veronii*), a Gram-negative, facultative anaerobic, rod-shaped bacterium, is widely distributed in freshwater and marine environments and has emerged as a major opportunistic pathogen in global aquaculture [7,8]. French microbiologist FW Hickman-Brenner first isolated *A. veronii* from the respiratory secretions of drowning in 1983 [9]. Subsequently, researchers have successfully isolated and identified this bacterium from various aquatic animals [8,10], including diverse fish species such as *Micropterus salmoides* [11], *Oreochromis niloticus* [12,13], *Ictalurus punctatus* [14], *Lateolabrax maculatus* [15], and hybrid sturgeon [16]. Notably, *A. veronii* is also considered an emerging zoonotic pathogen capable of causing acute gastroenteritis and extraintestinal infections in immunocompromised humans, highlighting its dual threat to public health and food safety [17,18]. In recent years, it has been reported domestically and internationally, with the epidemic displaying a clear upward trend and causing increasingly severe damage and economic losses [19,20,21]. The pathogenicity of *A. vernoii* is complex and heavily relies on synergistic repertoire determinants, including the *cytotonic enterotoxin* gene (*Act*), *aerolysin* (*Aer*), *heat-labile enterotoxin* (*Alt*), *lipase* (*Lip*), *outer membrane protein A* (*Omp*), and *exuperantia* gene (*Exu*) [21,22,23,24]. Additionally, the widespread and often injudicious application of antibiotics in aquaculture has drastically accelerated the selection of multidrug-resistant (MDR) strains [25].

Although *A. veronii* infection has been documented in a variety of commercial fish species, more data is required to determine its infection symptoms, pathogenic processes, and antibiotic susceptibility in different economic fish species in order to better prevent and treat such infections. Therefore, this study was the first to isolate and identify the core pathogenic pathogens from *P. variegatus* that had outbreaks of disease in an artificial breeding environment, assess the presence of virulence genes in them, determine their antibiotic sensitivity patterns using the Kirby–Bauer disk method, and verify the pathogenicity through artificial infection experiments [26]. These findings will provide a scientific basis for the in vitro selection of potential antimicrobial agents and the development of effective control strategies for *A. veronii* infections in aquaculture [27].

## 2. Results

### 2.1. Macroscopic External Examination of Diseased Paracobitis variegatus

**Figure 1:**
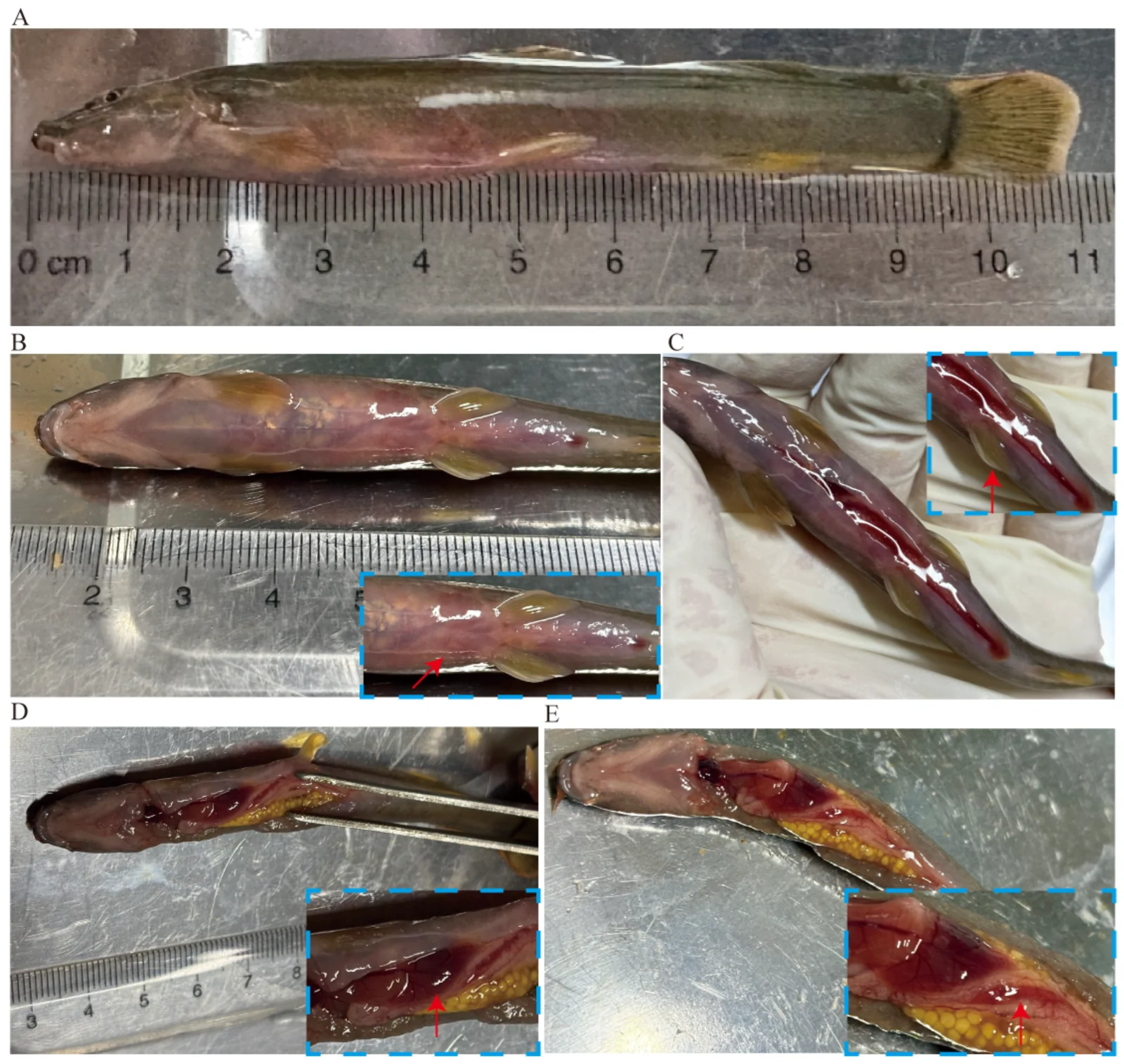
Clinical symptoms of diseased *Paracobitis variegatus*. (**A**) Lateral view of the intact fish (ruler marking in cm). (**B**) Close-up of the anterior region showing abdominal distension. (**C**) Preliminary dissection revealed massive ascites. (**D**,**E**) The liver was enlarged and congested with hemorrhagic lesions, and significant intestinal hemorrhage was also observed after dissection. The scarlet arrow signifies the site of the principal symptom. The blue dotted-line enclosure denotes an enlarged view of the main lesion at 2×.

The gross morphological and pathological features of the *P. variegatus* specimen are shown in Figure 1. Preliminary external examination revealed obvious abdominal distension accompanied by severe subcutaneous hyperemia in affected fish, with bright red hemorrhagic plaques visible through the translucent abdominal wall (Figure 1A,B). Postmortem dissection demonstrated abundant ascites within the coelomic cavity and extensive systemic hemorrhagic lesions, characterized by massive dark red blood clots, as well as hepatomegaly and hepatic hemorrhage (Figure 1C–E).

### 2.2. Isolation and Identification of Pathogenic Bacterium from Diseased Paracobitis variegatus

**Figure 2:**
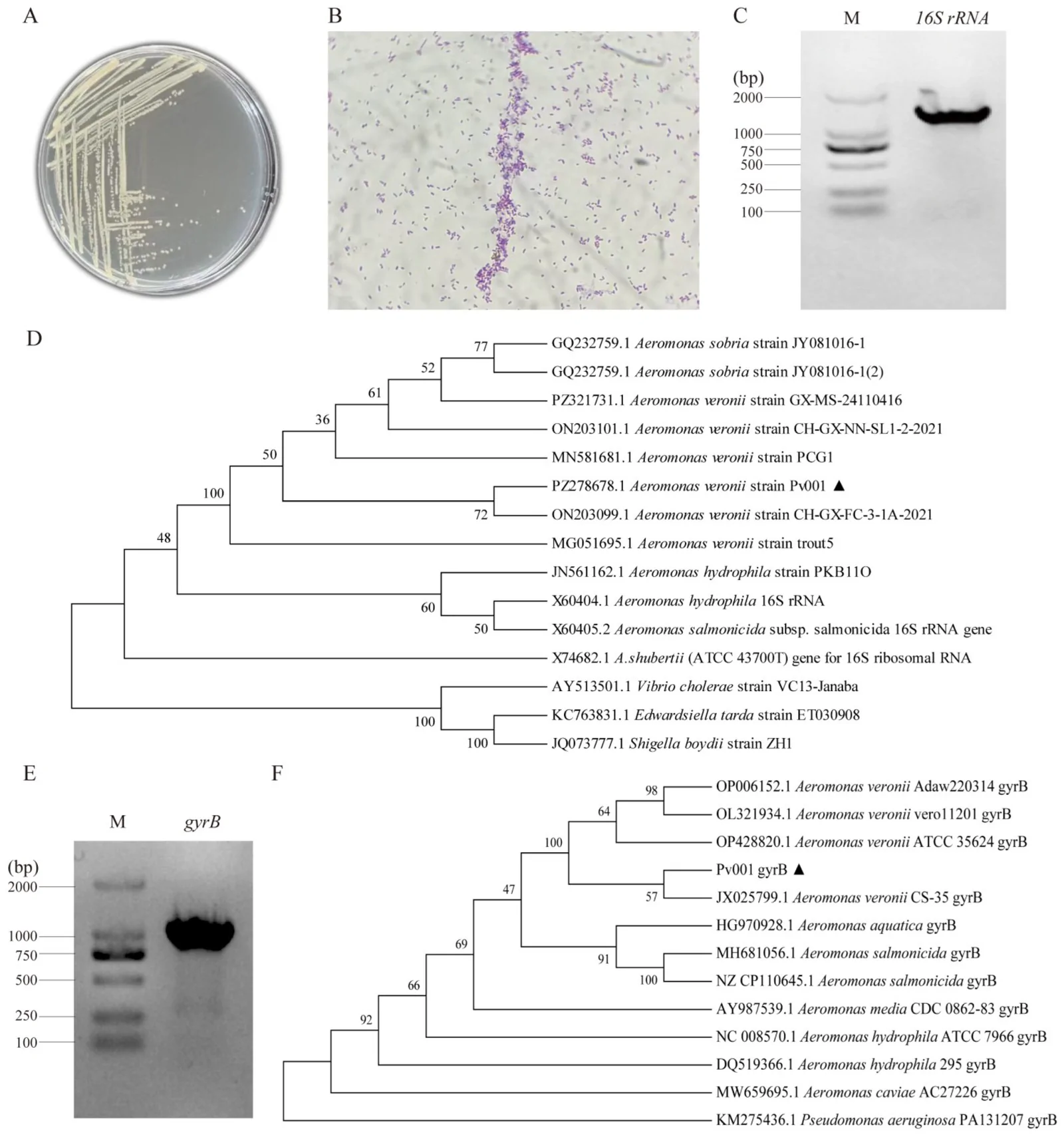
Identification of the pathogenic isolate strain. (**A**) Colonial morphology of the isolated strain on LB agar plates. (**B**) Gram staining of the pathogenic isolate strain. (**C**) Agarose gel electrophoresis of the PCR-amplified 16S rRNA gene. (**D**) Neighbor-joining phylogenetic tree based on 16S rRNA sequences showing the taxonomic position of the pathogenic isolate strain. (**E**) Agarose gel electrophoresis of the PCR-amplified gyrB gene. (**F**) Neighbor-joining phylogenetic tree based on gyrB sequences showing the taxonomic position of the pathogenic isolate strain. The black triangle (▲) indicates the isolated strain Pv001.

The colonies of strain Pv001 on Luria–Bertani (LB) agar plates were circular, smooth, milky-white, opaque, and slightly raised on the culture medium. Following Gram staining, the bacterial cells appeared as red, rod-shaped bacilli (Figure 2A,B), indicating that the isolate is a Gram-negative bacterium. The physiological and biochemical characteristics of the isolate are summarized in Table 1. The biochemical profile revealed 10 positive traits, including arginine decarboxylase, glucose, maltose, nitrate reduction, oxidase, peptone water, Simmons citrate, sucrose, β-Galactosidase, and Voges–Proskauer. Conversely, the isolate exhibited negative reactions for esculin, H_2_S production, lysine decarboxylase, ornithine decarboxylase, xylose, urea, and Methyl Red. Based on these phenotypic characteristics and in conjunction with the Bergey’s Manual of Systematic Bacteriology and Common Bacterial System Identification Manual, the isolated Pv001 was preliminarily identified as a member of the genus *Aeromonas*.

The 16S rRNA gene of strain Pv001 was successfully amplified *via* PCR and detected by agarose gel electrophoresis, yielding a specific amplicon of approximately 1465 bp (Figure 2C). BLAST homology alignment of the obtained sequence revealed that the 16S rRNA gene sequence of Pv001 shared 99% similarity with the sequences of *A. veronii* strains. Furthermore, the phylogenetic tree analysis (Figure 2D) demonstrated that strain Pv001 was clustered most closely with *A. veronii* strain CH-GX-FC-3-1A-2021.

The *gyrB* gene of strain Pv001 was successfully amplified *via* PCR and detected by agarose gel electrophoresis, yielding a specific amplicon of approximately 1065 bp (Figure 2E). BLAST homology alignment of the obtained sequence revealed that the *gyrB* gene sequence of Pv001 shared 99% similarity with the sequences of *A. veronii* strains. Furthermore, the phylogenetic tree analysis (Figure 2F) demonstrated that strain Pv001 was clustered most closely with *A. veronii* strain CS-35.

In summary, based on the colony morphology, physiological and biochemical characteristics, and 16S rRNA and *gyrB* gene sequencing analysis, the pathogenic isolate Pv001 was definitively identified as *A. veronii*.

### 2.3. Virulence Gene Detection and Antimicrobial Susceptibility Profiles

**Figure 3:**
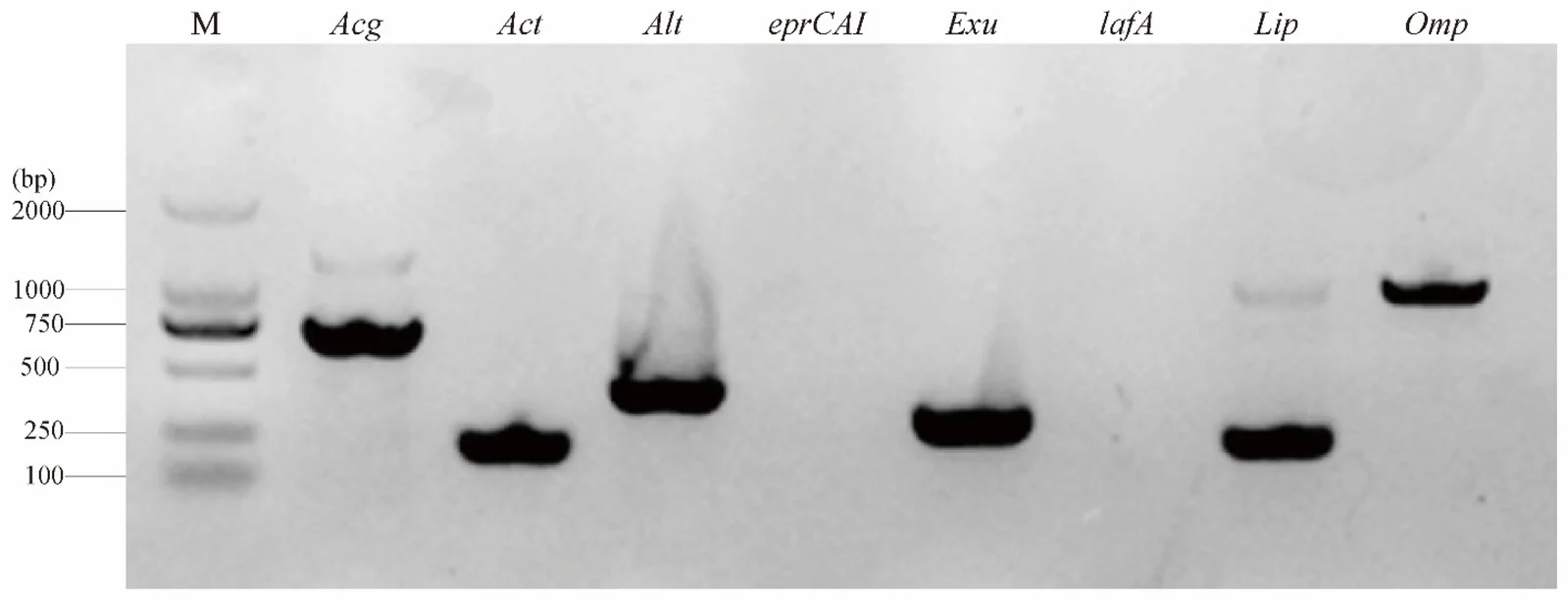
Agarose gel electrophoresis of the PCR-amplified virulence gene.

The virulence-associated genes of strain Pv001 were detected by PCR. Among the eight target virulence genes, six genes (*Acg*, *Act*, *Alt*, *Exu*, *Lip* and *Omp*) were successfully amplified from strain Pv001, yielding positive bands consistent with the expected fragment sizes. However, the other two genes (*eprCAI* and *lafA*) were nor detected (Figure 3).

Antimicrobial susceptibility of strain Pv001 to ten antimicrobial agents was determined using the Kirby–Bauer disk diffusion method. The results are summarized in Table 2. Ceftriaxone, chloramphenicol, ciprofloxacin, and tetracycline demonstrated the most substantial inhibitory zones against strain Pv001, measuring 35, 28, 27, and 24 mm, respectively. Trimethoprim-sulfamethoxazole generated moderate inhibitory zones measuring 16 mm, while erythromycin and gentamicin both produced zones of 15 mm. Conversely, lincomycin, penicillin, and ampicillin demonstrated no observable inhibition (0 mm).

### 2.4. Challenge Test Results

**Figure 4:**
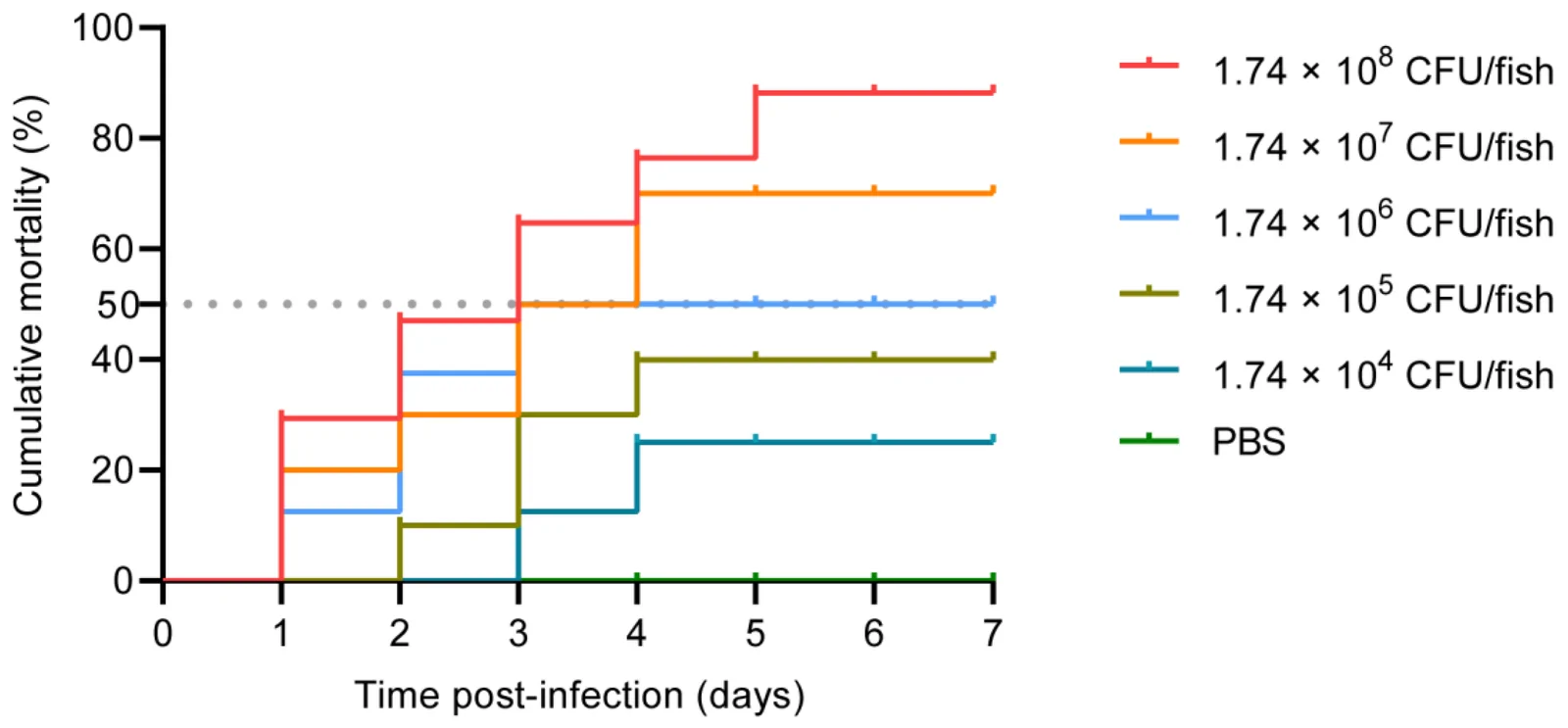
The cumulative mortality of *Paracobitis variegatus* infected with different concentrations of strain Pv001. Daily statistical mortality was calculated for 7 days, n = 50 for each group.

The pathogenicity of strain Pv001 was evaluated *via* artificial infection challenge. Challenged fish exhibited clinical signs consistent with those observed in the naturally diseased fish, including abdominal distension, severe ascites, and visceral hemorrhage, ultimately leading to mortality. The first death of the challenged *P. variegatus* occurred 7 h after *A. veronii* infection. On day 7, the cumulative mortality rates of *P. variegatus* infected with different concentrations of *A. veronii* were 88%, 70%, 50%, 40%, and 24% in groups of 1.74 × 10^8^, 1.74 × 10^7^, 1.74 × 10^6^, 1.74 × 10^5^, and 1.74 × 10^4^ CFU/fish, respectively (Figure 4). In contrast, the control group (PBS) remained healthy and exhibited no mortality throughout the experimental period. According to the gradient dose-challenge findings, the LD_50_ was determined to be 8.61 × 10^5^ CFU/fish utilizing SPSS’s Probit regression analysis technique (95% confidence interval: 3.32 × 10^5^ to 2.06 × 10^6^ CFU/fish). Furthermore, the pathogen was successfully re-isolated from moribund and dead challenged fish. The re-isolated strains were confirmed to be *A. veronii* based on morphological, biochemical, and 16S rRNA gene sequencing analyses, thereby fulfilling Koch’s postulates, showing it was the predominant pathogen driving the outbreak on the farm [28].

### 2.5. Histopathological Observation of Paracobitis variegatus

**Figure 5:**
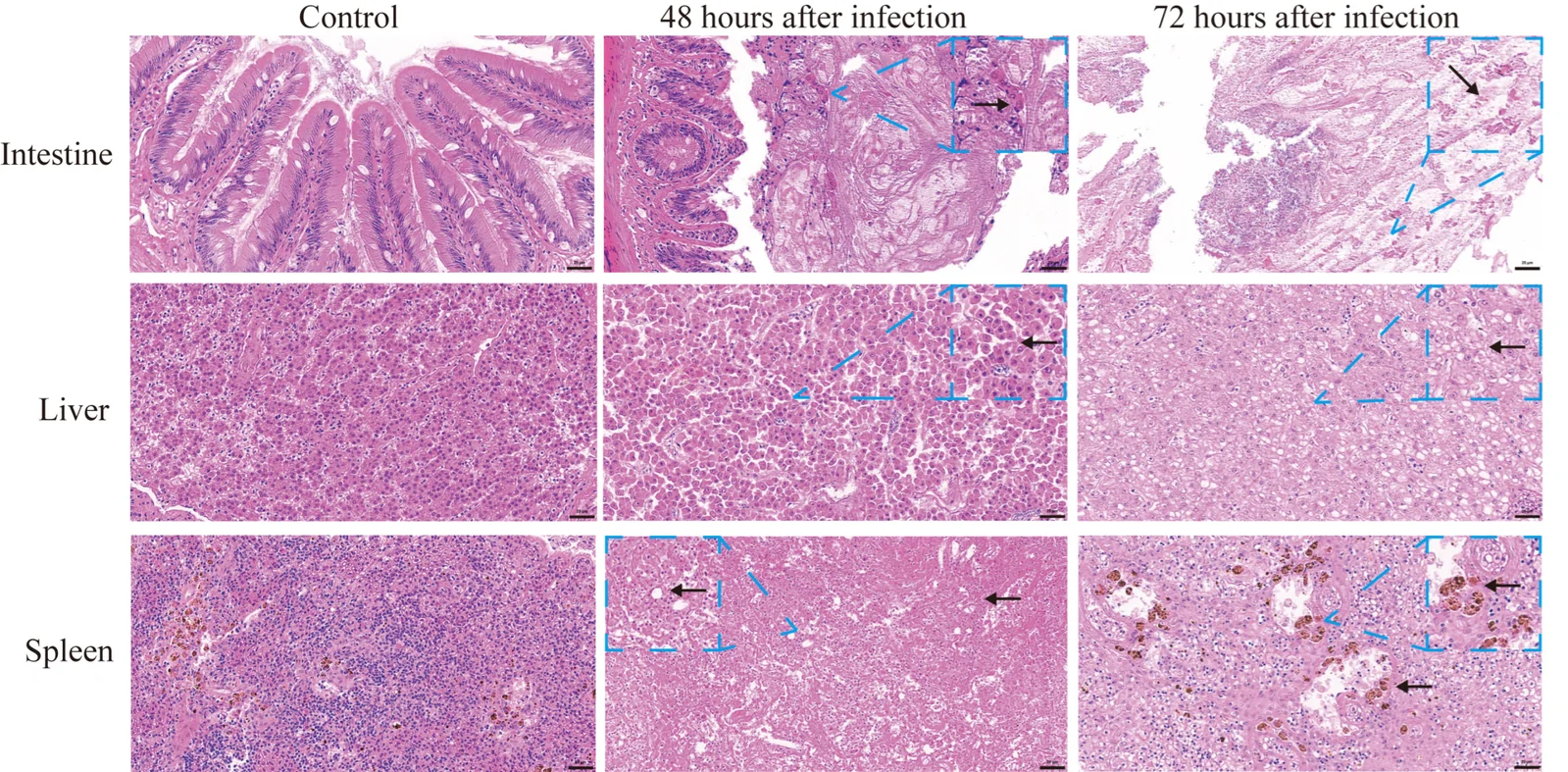
Histopathological changes in *Paracobitis variegatus* tissues (intestine, liver and spleen) after *A. veronii* challenge. The blue dotted-line rectangle shows a two-fold-enlarged picture of the pathogenic alterations. The black solid-line marker in the lower right corner signifies a measurement of 25 micrometers. The dark arrows signify the pathogenic alterations. Every image displays a view enlarged 60-fold.

Histopathological examination further revealed severe tissue lesions in the challenged fish (Figure 5). In the intestinal tissues, the control group exhibited intact intestinal villi and mucosal structures. However, evident shedding of the epithelial desquamation was observed at 48 h post infection (indicated by black arrows), progressing to extensive necrosis and disintegration of the mucosal layer by 72 h. In the liver, hepatocytes were arranged uniformly in the control group. Vacuolation and cellular swelling of hepatocytes were observed at 48 h post infection (black arrows), while severe focal necrosis, structural blurring, congestion, and karyolysis were present at 72 h. In the spleen, normal architecture with distinct boundaries between the red and white pulp was observed in the control group. Focal hemorrhage and structural disruption of splenic cells were noted at 48 h post infection (black arrows), progressing to severe focal necrosis with a marked aggregation of MMCs at 72 h. Collectively, these findings demonstrated that strain Pv001 induces severe multiorgan pathological damage and exerts a significant lethal effect on the host.

## 3. Discussion

In the present study, a predominant pathogenic bacterial strain, Pv001, was initially derived from naturally affected *P. variegatus*. Based on its colony morphology, Gram-negative traits, biochemical profiles, and 16S rRNA gene phylogenetic assessment, the solitary strain was conclusively identified as *Aeromonas veronii*. The 16S rRNA gene sequence of strain Pv001 demonstrated a 99% match to *A. veronii* strains in the NCBI GenBank database, and phylogenetic tree analysis validated its tight association with *A. veronii*. These results correspond with earlier reports about Aeromonas spp. extracted from several aquaculture fish [29]. It is important to highlight that earlier investigations have demonstrated that, despite the utility of 16S rRNA sequencing for genus identification, comparative analyses suggest that its resolution is often inadequate for distinguishing closely related species [29,30]. Thus, to further verify whether this strain is *A. veronii*, we also identified the distinctive housekeeping gene *gyrB* [18,31]. The results demonstrated that the *gyrB* gene sequence of strain Pv001 showed more than 98% similarity to that of *A. veronii* in the NCBI GenBank database, thereby validating that this strain is indeed *A. veronii*. In summary, the integration of biochemical profiles, colony morphology, and the significant similarity in the 16S rRNA and *gyrB* gene sequences in this research offered compelling evidence to definitively classify Pv001 as *A. veronii*.

The virulence gene composition of strain Pv001 demonstrated the existence of six of the eight examined genes (*Acg*, *Act*, *Alt*, *Exu*, *Lip*, and *Omp*). Among these, *act* and *alt* are thoroughly defined principal virulence determinants that can provoke intestinal fluid secretion and elicit host inflammatory reactions [32]. The *Lip* gene encodes for lipase, which aids in the breakdown of host cell membranes, thereby enhancing bacterial invasion and widespread distribution within the host [33]. Conversely, the failure to detect the flagellar subunit *lafA* and the serine protease *eprCAI* in Pv001 suggests that the strain may rely on alternative virulence genes to produce illness. It should be noted that due to the unavailability of positive control strains, these negative amplification results cannot definitively confirm the complete absence of these genes. However, given this strain’s high pathogenicity, it is plausible that it possesses additional genes that impact flagellar motility, which is consistent with findings of certain environmental *A. veronii* strains [34]. Our results exhibit partial resemblance to those documented by other researchers. In largemouth bass, both *Act* and *Aer* were identified [35]. Our findings suggest that the Pv001 strain may damage the host cell membrane through *Lip*, leading to the discharge of cellular intestinal fluid and an inflammatory reaction mediated by the *Act* and *Alt* virulence genes. Considering the primary symptoms noted in naturally infected *P. variegatus*, we can plausibly conjecture that this could be the underlying explanation for the considerable buildup of ascites and the inflammatory reaction we witnessed. Certainly, additional evidence is needed to substantiate this claim [31], through, for example, ascertaining the genomic data of Pv001, investigating the virulence genes it harbors, and performing comprehensive studies on the interplay between these virulence genes and the host immune response—this is exactly what we want to undertake next. Moreover, the composition and total number of virulence genes identified in the isolated strains from different environments may vary [36]. The extensive prevalence of virulence-related genes in aquatic pathogens emphasizes the intricacy of Aeromonas pathogenicity and reinforces the necessity for ongoing genomic monitoring [37].

Antimicrobial susceptibility analysis of Pv001 strain showed different susceptibility profiles against routinely used antibacterials. The absence of quantifiable inhibitory zones for penicillins and lincomycin, coupled with moderate zones for erythromycin and sulfamethoxazole-trimethoprim, corresponds with other research results [11,38]. Nonetheless, it is crucial to highlight that the CLSI M100 (35th edition) clearly indicates disk-shaped diffusion has not been thoroughly examined or endorsed for *Aeromonas spp.*, due to the absence of species-specific zone diameter breakpoints. Consequently, our findings are presented solely as in vitro assessments. It is essential to acknowledge that standard quality-control (QC) strains were not accessible in our laboratory throughout this investigation, which further constrains the rigorous interpretation of these disk diffusion outcomes. The absence of inhibitory zones for β-lactams (penicillin and ampicillin) may correlate with the inherent capacity of Aeromonas species to generate chromosomal β-lactamases [39,40]. Despite the presence of mild zones for sulfonamides, the extensive historical application of these compounds in aquaculture settings probably leads to a significant prevalence of resistance genes [41,42,43]. This research exclusively delineated the phenotypic characteristics, although the results strongly advise against the extensive use of penicillins and sulfonamides in regional *P. variegatus* aquaculture. This corresponds with the One Health viewpoint, as unregulated antibiotic application in aquatic environments can facilitate the spread of antimicrobial resistance (AMR). To overcome the existing constraints of CLSI M100 and thoroughly clarify the molecular foundations of these phenotypes, forthcoming research should integrate standardized MIC testing, define precise epidemiological cutoff values (ECVs) for *A. veronii*, and employ whole-genome sequencing to uncover the fundamental resistance determinants.

Artificial infection experiments successfully reproduced the clinical signs observed in naturally diseased fish, including abdominal distension, severe ascites and visceral hemorrhage, strictly fulfilling Koch’s postulates and confirming Pv001 as the causative agent [44]. The regression infected results revealed that the 50% fatal dose of *P. variegatus* was only 8.61 × 10^5^ CFU/fish, with the first death occurring in the challenge group 7 h after infection. In general, extremely pathogenic *Aeromonas* infections have an LD_50_ of between 10^4^ and 10^5^ CFU per fish [45]. It should be noted that this intraperitoneal challenge route bypasses the natural mucosal barriers of the fish, which may result in a relatively lower LD_50_ compared to immersion or oral challenge methods. Compared with published data, our calculated LD_50_ is slightly higher; however, considering the severe clinical symptoms and high mortality induced, strain Pv001 still exhibits high virulence to *P. variegatus* [45]. Furthermore, it is important to clarify that the specific dose of 1.74 × 10^6^ CFU/fish used for histopathological sampling was a pre-determined challenge dose based on preliminary trials, rather than the calculated LD_50_. Histopathological analysis further revealed severe multiorgan damage. In the intestine, severe epithelial desquamation at 48 h and extensive necrosis by 72 h were observed, which severely impairs nutrient absorption and compromises the mucosal barrier function. Similar inflammatory lesions and mucosal shedding were reported in intestinal tissues of common carp and channel catfish during *Aeromonas* infections [46,47]. In the liver, hepatocytes exhibited significant vacuolar degeneration and swelling at 48 h, progressing to severe focal necrosis, congestion, and karyolysis at 72 h. This is supported by the research on rainbow trout, which found significant vacuolar degeneration in the livers of rainbow trout infected with *Acinetobacter johnsonii* [48]. Meanwhile, the spleen displayed structural disorganization and marked aggregation of MMCs at 72 h post infection. MMCs are unique phagocytic structures in teleost fish that play a critical role in antigen trapping and immune defense during bacterial invasion [49]. These results demonstrate that Pv001 is a highly invasive and cytotoxic pathogen capable of inducing acute systemic septicemia in *P. variegatus*.

## 4. Materials and Methods

### 4.1. Diseased Sample Collection

A fish farm in Xichang City, Sichuan Province, China, primarily raised *P. variegatus* in round breeding tanks. In November 2025, following several consecutive sunny days, the farm had a sudden mass die-off of *P. variegatus*. The dead *P. variegatus* weighed between 12 and 30 g and measured 10 to 20 cm long. The dead fish’s skin was bleeding in spots or patches, the fin bases were congested, and the abdomen was clearly enlarged. Finally, the death rate of *P. variegatus* on the farm reached approximately 45%. To determine the pathogen causing this outbreak, 20 visibly diseased *P. variegatus* were chosen at random and quickly sent to the Key Laboratory of Animal Disease Detection and Prevention at Panxi of Xichang University. This sampling procedure exclusively entailed the acquisition of naturally afflicted and deceased fish for clinical assessment and pathogen identification, devoid of any experimental manipulation of living specimens.

### 4.2. Bacteria Isolation and Culture

Diseased fish with recognizable clinical symptoms and those in imminent danger of dying were selected. An MS-222 solution at 80 mg/L (R03050, Leagene, Beijing, China) was utilized to induce anesthesia, thereafter conducting bacterial isolation. The body surface of each fish was disinfected thoroughly with cotton balls soaked in 75% ethanol under aseptic conditions prior to dissection. Small tissue samples were collected from obviously lesioned intestines, livers, and spleens using a sterile inoculating loop, and streaked onto LB agar plates. After incubation at 28 °C for 20 h, single colonies were picked for purification, and colonial morphology was observed. Dominant single colonies with consistent shape, size and pigmentation were re-streaked onto fresh LB medium for repeated purification. The purified primary isolate was tentatively designated strain Pv001, and its colonial and cellular morphological features were characterized subsequently. The purified strain was preserved at 4 °C for subsequent assays.

### 4.3. Morphological, Physiological and Biochemical Identification

Gram staining of the purified strain Pv001 was performed using a commercial Gram Stain Kit (Solarbio, Beijing, China) according to the manufacturer’s instructions. Bacteria morphology, size, and staining characteristics were observed and photographed under a light microscope. For physiological and biochemical tests, the purified pathogen was streaked onto LB agar plates and incubated overnight at 28 °C. Biochemical characterization was carried out using bacterial micro-biochemical reaction tubes (Hangzhou Microbial Reagent. Co., Ltd., Hangzhou, China) with reference to Bergey’s Manual of Systematic Bacteriology and the Common Bacterial System Identification Manual. The Pv0001 strain was introduced into MR-VP biochemical test tubes (Solarbio, Beijing, China) and incubated at 28 °C for 48 h to conduct the Methyl Red test and Voges–Proskauer reaction.

### 4.4. 16S rRNA and gyrB Gene Sequencing and Phylogenetic Analysis

Fresh bacterial suspension was used as the template for Polymerase Chain Reaction (PCR), amplification of the 16S rRNA with the universal primer pair 27F and 1492R, and amplification of the gyrB gene with the specific primer pair gyrB-F and gyrB-R (Table S1). The PCR thermal cycling program was set as follows: initial denaturation at 94 °C for 10 min, 30 cycles of denaturation at 94 °C for 1 min, annealing at 55 °C for 1 min, extension at 72 °C for 2 min, and final extension step at 72 °C for 10 min, followed by incubation at 4 °C for temporary storage. An aliquot of 5 μL PCR product was separated *via* 1% agarose gel electrophoresis to verify target amplicons. The PCR products of the positive sample were sent to Sangon Biotech (Shanghai, China) Co., Ltd. for bidirectional sequencing. The obtained 16S rRNA and gyrB sequence of the isolate was subjected to BLASTn homology alignment against published sequences of the genus *Aeromonas* deposited in the GenBank database. The neighbor-joining (NJ) phylogenetic tree was constructed using MEGA software (version 11.0, Sudhir Kumar, PA, USA) with 1000 bootstrap replications. The finalized 16S rRNA nucleotide sequence of the strain was submitted to the NCBI GenBank database (submission number: SUB16121373).

### 4.5. Detection of Virulence Genes

Based on previous studies, PCR assays were performed to screen the presence of eight virulence-related genes (*Acg*, *Act*, *Alt*, *eprCAI*, *Exu*, *lafA*, Lip, *Omp*) in isolate Pv001 [50]. The primer sequences and expected amplicon sizes for each target virulence gene are summarized in Table S1. The PCR mixture and thermal cycling conditions were similar to those described in Section 4.4. Sterile ultrapure water was used as a negative control in each PCR run. Amplified PCR products were visualized *via* agarose gel electrophoresis. It should be noted that positive control strains (e.g., *A. veronii* strains known to harbor the *eprCAI* and *lafA* genes) were unavailable in our laboratory.

### 4.6. Antimicrobial Susceptibility Testing

Antimicrobial susceptibility of Pv001 was determined via the Kirby–Bauer (K-B) disk diffusion method in accordance with standard microbiological protocols. Briefly, a bacterial suspension of strain Pv001 was prepared, and 100 μL of the suspension was spread evenly onto LB agar plates using a sterile disposable spreader. Then, 10 types of antimicrobial susceptibility disks were placed separately on the agar surface with sterile forceps. The plates were incubated aerobically at 28 °C in an inverted position for 18 h, followed by measurement of the diameter of each inhibition zone. All assays were performed in three biological replicates to define the susceptibility profile of the isolate against each tested antimicrobial agent. The CLSI M100 (35th edition) does not endorse disk diffusion for *Aeromonas* spp.; owing to the absence of verified species-specific zone diameter breakpoints, the findings were presented solely as the recorded inhibitory zone diameters without designating clear susceptible or resistant classifications.

### 4.7. Pathogenicity Artificial Infection Assay

Healthy *P. variegatus* (average length 18.5 ± 1.5 cm and weight 26.5 ± 3.5 g) were obtained from a local aquaculture farm in Xichang City, Sichuan Province, China, which had no reported bacterial disease outbreaks in the preceding two years. Fish were acclimated in laboratory tanks for a fortnight under regulated parameters (temperature 22 ± 0.5 °C, pH 7.2 ± 0.3, dissolved oxygen > 5.0 mg/L, ammonia nitrogen < 0.2 mg/L) and provided a commercial feed amounting to 3% of their body mass each day. Before the challenge, three fish were selected at random and anesthetized with MS-222. Their liver, kidney, and spleen tissues were collected and pathogenic bacteria were isolated *via* streaking on LB agar plates. Then, 20 circular single colonies were randomly selected from each plate, and the 16S rRNA and gyrB genes were amplified using the PCR method described in Section 4.4 to identify *A. veronii*. As a result, no *A. veronii* were identified. The subsequent studies involved solely fish exhibiting healthy locomotor behavior and devoid of skin lesions. This animal study commenced on 20 March 2026, and received authorization from the Animal Ethics Committee of Xichang University (Sichuan, China).

A total of 300 healthy fish were randomly distributed into six groups (n = 50 for each group), consisting of five experimental infection groups and one control group. Each group was additionally partitioned into two independent replicate tanks, accommodating 25 fish per tank. All the fish were reared in the previously mentioned conditions within uniform plastic tanks. Following acclimation, fish in the five experimental groups received intraperitoneal injections of Pv001 live culture at dosages of 1.74 × 10^8^, 1.74 × 10^7^, 1.74 × 10^6^, 1.74 × 10^5^, and 1.74 × 10^4^ CFU per fish, respectively. To eradicate experimental interference, fish in the blank control groups received 0.1 mL of sterile phosphate-buffered solution (PBS) for each fish under identical operational conditions.

After the infection, all fish were meticulously observed for a period of seven days. Fish in a moribund state, marked by balance loss, lethargy, or insensitivity to stimuli, were gently put to death using an excessive concentration of MS-222 (500 mg/L) to reduce distress. Additionally, any dead fish were immediately removed from the tanks to prevent water fouling and cannibalism and recorded. Daily deaths were noted for every group. Utilizing the gradient dose-challenge data, the LD_50_ was established through Probit regression analysis with SPSS software (version 27.0).

To rigorously investigate the histopathological changes, the optimum experimental challenge dose of 1.74 × 10^6^ CFU/fish was established based on preliminary experiments. This dosage was selected as it elicited typical clinical manifestations without resulting in imminent widespread mortality. At 48 and 72 h after infection, three severely infected fish with typical symptoms were randomly selected from the 1.74 × 10^6^ CFU/fish group, while three fish were simultaneously and randomly selected from the control group at each respective period (n = 3 per group per time point). This sample was conducted simultaneously with the mortality surveillance. The intestine, liver, and spleen tissues were acquired for germ re-isolation as described in Section 4.2, and for ensuing histological analysis.

### 4.8. Histological Analysis with Hematoxylin and Eosin (H&E) Staining

The diseased tissue samples from the infected fish were generated utilizing the technique described in the previously published study [51]. In summary, intestinal, hepatic, and splenic tissues were harvested from dying fish in the 1.74 × 10^6^ CFU/fish group and the control group at 48 and 72 h after infection (n = 3 for each group at each time interval), as stated in Section 4.7. All tissue samples were promptly fixed in 4% paraformaldehyde for 24 h at ambient temperature. After fixation procedures, all tissue samples underwent gradient dehydration through a sequence of ethanol solutions (70%, 85%, 95%, and 100%) for 5 min at each concentration. Subsequent to xylene clearance, the specimens were encased in paraffin and sliced serially to a thickness of 5 μm. The acquired sections were dyed utilizing a hematoxylin and eosin staining kit (Beyotime, Shanghai, China) in precise adherence to the manufacturer’s instructions. Histopathological alterations were noted and captured with a ZEISS Axio 5 light microscope (Zeiss, Jena, Germany).

## 5. Conclusions

In summary, *A. veronii* Pv001 was identified as a primary bacterial pathogen associated with septicemia outbreak in *P. variegatus*. Virulence gene screening revealed that Pv001 harbored multiple virulence-related genes, including *Acg*, *Act*, *Alt*, *Exu*, *Lip*, and *Omp*. In vitro antimicrobial assays revealed that ceftriaxone, chloramphenicol, ciprofloxacin, and tetracycline displayed considerable inhibitory effects on Pv001, but ampicillin, penicillin, and lincomycin exhibited no discernible inhibitory activity. Artificial challenge confirmed the high virulence of strain Pv001, which induced acute mortality and severe multiorgan lesions (intestine, liver, and spleen), thereby fulfilling Koch’s postulates. These findings provide in vitro references for potential antimicrobial selection and a foundation for future in vivo efficacy trials and mechanistic investigations.

## Figures and Tables

**Table 1:** Physiological and biochemical identification of isolated strains.

Biochemical Item	Result	Biochemical Item	Result
Arginine decarboxylase	+	Peptone water	+
Esculin	−	Xylose	−
Glucose	+	Simmons citrate	+
H_2_S production	−	Sucrose	+
Lysine decarboxylase	−	Urea	−
Maltose	+	β-Galactosidase	+
Nitrate reduction	+	Methyl Red	−
Ornithine decarboxylase	−	Voges–Proskauer	+
Oxidase	+		

“+” means positive; “−“ means negative.

**Table 2:** Antimicrobial susceptibility profiles of the isolated strain determined by the Kirby–Bauer disk diffusion method.

Antibacterial Drugs	Antimicrobial Agent	Content (μg/disk)	Measured Zone Diameter (mm)
Macrolides	Erythromycin	15	15
Sulfonamides	Trimethoprim- sulfamethoxazole	23.75/1.25	16
Phenicols	Chloramphenicol	30	28
Aminoglycosides	Gentamicin	10	15
Fluoroquinolones	Ciprofloxacin	5	27
Lincosamides	Lincomycin	2	0
Tetracyclines	Tetracycline	30	24
β-lactams	Penicillin	10	0
	Ampicillin	10	0
	Ceftriaxone	30	35

## Data Availability

The original contributions presented in this study are included in the article/Supplementary Materials. Further inquiries can be directed to the corresponding author.

## Supplementary Materials

The following supporting information can be downloaded at https://www.mdpi.com/article/10.3390/ijms27219776/s1.
