# Supplementary material for: Isolation, Identification, and Pathogenicity of Aeromonas veronii from Diseased Paracobitis variegatus
Source: Int J Mol Sci. 2026 Oct 30;27(21):9776. doi: 10.3390/ijms27219776 (PMC13762927; doi:10.3390/ijms27219776)
Supplement: Supplementary file 1 [file ijms-27-09776-s001.zip › ijms-4620676-supplementary.pdf]

Table S1 The primers were used in this study

Gene symbols	Primers	Sequences (5′-3′)	Product size (bp)
16S rRNA	27F	AGAGTTTGATCCTGGCTCAG	1 500
	1492R	GGTTACCTTGTTACGACTT	
gyrB	gyrB-F	GAAGTCATCATGACCGTTCTGCA	1047
	gyrB-R	AGCAGGGTACGGATGTGCGAGCC	
Acg	Acr-F	AACAAGCACCCGTTAAGCCAC	761
	Acr-R	ACGTAGTCGAGCCCCTTGAGG	
Act	Act-F	AGAAGGTGACCACCAAGAACA	232
	Act-R	AACTGACATCGGCCTTGAACTC	
Alt	Alt-F	TGACCCAGTCCTGGCACGGC	442
	Alt-R	GGTGATCGATCACCACCAGC	
eprCAI	eprCAI-F	GCTCGACGCCCAGCTCACC	1026
	eprCAI-R	GGCTCACCGCATTGGATTCG	
Exu	Exu-F	AGACATGCACAACCTCTTCC	323
	Exu-R	GATTGGTATTGCCTTGCAAC	
lafA	lafA-F	GGTCTGCGCATCCAACTC	550
	lafA-R	GCTCCAGACGGTTGATG	
Lip	Lip-F	ATCTTCTCCGACTGGTTCGG	247
	Lip-R	CCGTGCCAGGACTGGGTCTT	
OmpAI	OmpAI-F	GACGATATCATGATGAAAATGGCTCTT	1026
	OmpAI-R	GCGAAGCTTTTACTTCTGAACTTCTTG	
